# P-311. Real-world Persistence to Long-acting Cabotegravir Versus Oral PrEP for HIV Prevention in Participants Using a Digital Health Companion Tool (AURORA Study)

**DOI:** 10.1093/ofid/ofaf695.530

**Published:** 2026-01-11

**Authors:** W David D Hardy, Angela D Settle, Kelly E Pillinger, Leah Molloy, Laura Simone, Chris Napolitan, Chelsie Chadha, Melissa Rodriguez, Jeffrey D Carter

**Affiliations:** Keck School of Medicine of the University of Southern California, Los Angeles, California; West Virginia Health Right, Inc., Charleston, West Virginia; PRIME Education, New York, NY; PRIME Education, New York, NY; PRIME Education, LLC, Fort Lauderdale, Florida; PRIME Education, New York, NY; PRIME Education, New York, NY; PRIME Education, New York, NY; PRIME Education, LLC, Fort Lauderdale, Florida

## Abstract

**Background:**

Long-acting injectable cabotegravir (CAB-LA) was shown to be more effective than daily oral PrEP in the HPTN 083 and 084 studies, primarily driven by inadequate adherence to oral PrEP. This pilot study examined rates of persistence to CAB-LA versus oral PrEP in users engaged in a digital health tool.

**Figure:**
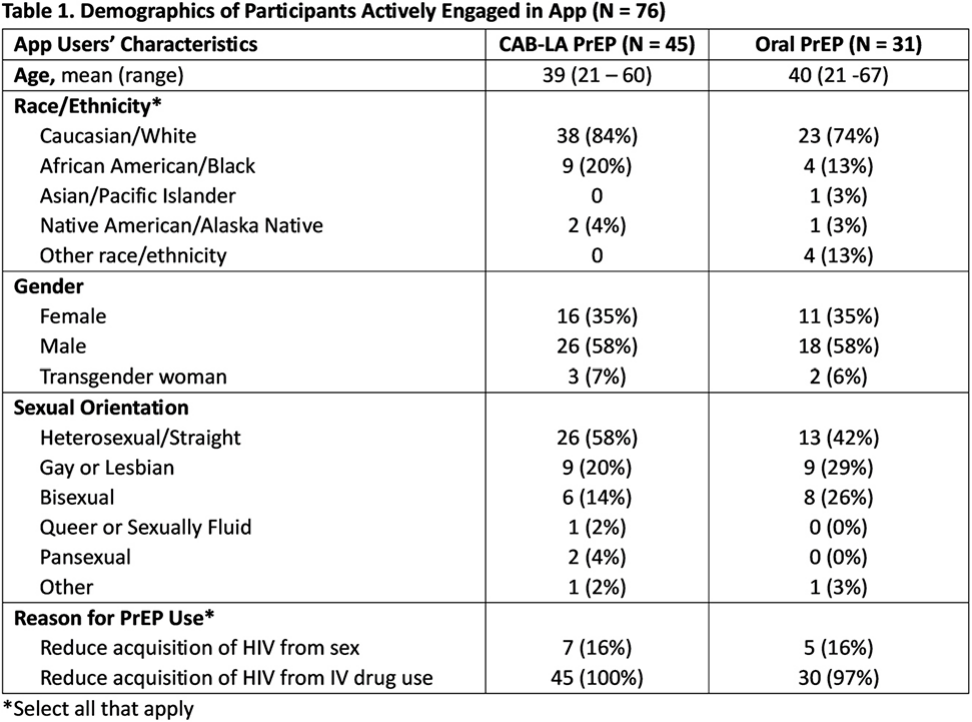
Demographics of Participants Actively Engaged in App (N = 76)

**Figure:**
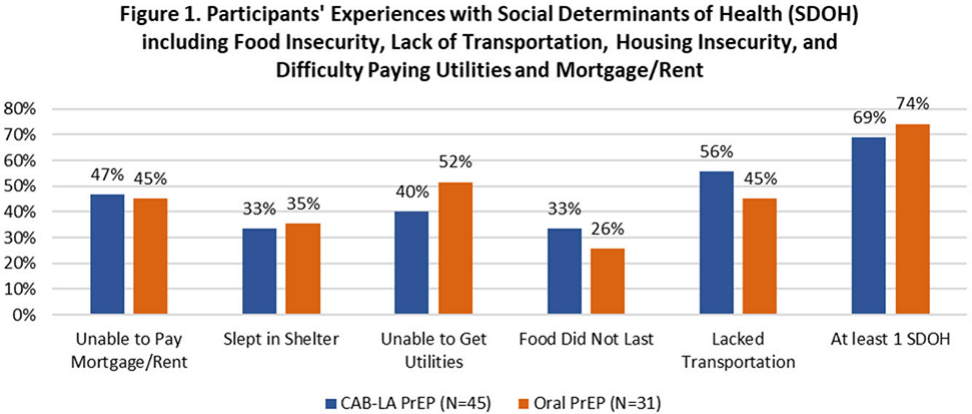
Participants' Experiences with Social Determinants of Health (SDOH) including Food Insecurity, Lack of Transportation, Housing Insecurity, and Difficulty Paying Utilities and Mortgage/Rent

**Methods:**

Participants (≥ 18 years old) prescribed CAB-LA, daily FTC/TDF, or daily FTC/TAF for HIV prevention by providers at West Virginia Health Right in Charleston, WV and with access to a smartphone were eligible for enrollment (October 2023-November 2024) in the 1-year study. Participants received access to a customized digital health app through Reciprocity Health® that included reminders to take PrEP and get labs, PrEP education, and surveys about social determinants of health (SDOH) and experiences with PrEP. Modest financial incentives encouraged users to reach milestones. Primary endpoint of persistence at 3 months was analyzed using chi-square tests.

**Results:**

There were 123 participants enrolled (N=75 CAB-LA; N = 48 oral PrEP) with 76 (62%) actively engaged to date in the digital app (N=45 CAB LA; N = 31 oral PrEP) with 58% male, 51% heterosexual, and 17% Black (Table 1). Additionally, 71% of app users reported experiencing at least 1 SDOH, with 58% experiencing 2 or more, with limited access to transportation (51%) most common (Fig 1). The primary endpoint of persistence at month 3 was 67% for the CAB-LA group vs. 55% for the oral PrEP group (p = .30). The primary reasons for PrEP discontinuation among those who reported were stopping injectable substance use for CAB-LA users and consistent condom use and regular HIV testing for oral PrEP users. In the first 3 months, on average 34% of oral PrEP users reported missing at least 1 dose per week. Adverse effects (AE) at month 3 were reported by 32% of CAB-LA users vs. 13% of oral PrEP users (p = .10). Of those with AE, 11/11 CAB-LA users felt they were acceptable or indifferent whereas 1/3 oral PrEP users felt they were unacceptable. No new HIV diagnoses occurred in either group.

**Conclusion:**

CAB-LA users who engaged with a digital health app demonstrated numerically higher rates of persistence at month 3 compared to oral PrEP users. High rates of SDOH across the study population underscore the need to address these factors in groups benefiting from HIV prevention.

**Disclosures:**

W David D. Hardy, MD, Gilead Sciences: Advisor/Consultant|Merck: Advisor/Consultant|ViiV Healthcare: Advisor/Consultant

